# Group B *Streptococcus* early-onset disease and observation of well-appearing newborns

**DOI:** 10.1371/journal.pone.0212784

**Published:** 2019-03-20

**Authors:** Alberto Berardi, Caterina Spada, Maria Letizia Bacchi Reggiani, Roberta Creti, Lorenza Baroni, Maria Grazia Capretti, Matilde Ciccia, Valentina Fiorini, Lucia Gambini, Giancarlo Gargano, Irene Papa, Giancarlo Piccinini, Vittoria Rizzo, Fabrizio Sandri, Laura Lucaccioni, on behalf of the GBS Prevention Working Group of Emilia-Romagna

**Affiliations:** 1 Terapia Intensiva Neonatale, Dipartimento Integrato Materno-Infantile, Azienda Ospedaliero-Universitaria Policlinico, Modena, Italy; 2 Medico in formazione, Scuola di Specializzazione in Pediatria, Università degli Studi di Modena e Reggio, Modena, Italy; 3 Dipartimento di Medicina Specialistica, Diagnostica e Sperimentale, Azienda Ospedaliero-Universitaria S.Orsola-Malpighi—Università di Bologna, Bologna, Italy; 4 Reparto di Antibiotico Resistenza e Patogeni Speciali (AR-PS) Dipartimento di Malattie Infettive Istituto Superiore di Sanità, Roma, Italy; 5 Terapia Intensiva Neonatale, Dipartimento Ostetrico e Pediatrico, Istituto di Ricovero e Cura a Carattere Scientifico IRCCS, Arcispedale Santa Maria Nuova, Reggio Emilia, Italy; 6 Terapia Intensiva Neonatale, Dipartimento Del Bambino, Della Donna e Delle Malattie Urologiche, Azienda Ospedaliero-Universitaria Sant’Orsola–Malpighi, Bologna, Italy; 7 Terapia Intensiva Neonatale, Dipartimento Materno Infantile, Ospedale Maggiore, Bologna, Italy; 8 Pediatria, Ospedale B Ramazzini, Carpi, Italy; 9 Terapia Intensiva Neonatale, Azienda Ospedaliero-Universitaria Policlinico, Parma, Italy; 10 Terapia Intensiva Neonatale, Ospedale Infermi, Rimini, Italy; 11 Pediatria, Ospedale Santa Maria Delle Croci, Ravenna, Italy; 12 Terapia Intensiva Neonatale e Pediatrica, Ospedale Civile M. Bufalini, Cesena, Italy; Centre Hospitalier Universitaire Vaudois, FRANCE

## Abstract

**Background:**

International guidelines lack a substantial consensus regarding management of asymptomatic full-term and late preterm neonates at risk for early-onset disease (EOS). Large cohorts of newborns are suitable to increase the understanding of the safety and efficacy of a given strategy.

**Methods:**

This is a prospective, area-based, cohort study involving regional birth facilities of Emilia-Romagna (Italy). We compared cases of EOS (at or above 35 weeks’ gestation) registered in 2003–2009 (baseline period: 266,646 LBs) and in 2010–2016, after introduction of a new strategy (serial physical examinations, SPEs) for managing asymptomatic neonates at risk for EOS (intervention period: 265,508 LBs).

**Results:**

There were 108 cases of EOS (baseline period, n = 60; intervention period, n = 48). Twenty-two (20.4%) remained asymptomatic through the first 72 hours of life, whereas 86 (79.6%) developed symptoms, in most cases (52/86, 60.5%) at birth or within 6 hours. The median age at presentation was significantly earlier in the intrapartum antibiotic prophylaxis (IAP)-exposed than in the IAP-unexposed neonates (0 hours, IQR 0.0000–0.0000 vs 6 hours, IQR 0.0000–15.0000, p<0.001). High number of neonates (n = 531) asymptomatic at birth, exposed to intrapartum fever, should be treated empirically for each newborn who subsequently develops sepsis. IAP exposed neonates increased (12% *vs* 33%, p = 0.01), age at presentation decreased (median 6 *vs* 1 hours, p = 0.01), whereas meningitis, mechanical ventilation and mortality did not change in baseline vs intervention period. After implementing the SPEs, no cases had adverse outcomes due to the strategy, and no cases developed severe disease after 6 hours of life.

**Conclusions:**

Infants with EOS exposed to IAP developed symptoms at birth in almost all cases, and those who appeared well at birth had a very low chance of having EOS. The risk of EOS in neonates (asymptomatic at birth) exposed to intrapartum fever was low. Although definite conclusions on causation are lacking, our data support SPEs of asymptomatic newborns at risk for EOS. SPEs seems a safe and effective alternative to laboratory screening and empirical antibiotic therapy.

## Introduction

Group B *Streptococcus* (GBS) is a leading cause of neonatal sepsis in high-income countries.[1] GBS early-onset sepsis (EOS) results from mother-to-infant transmission at delivery. Most neonates with EOS are symptomatic at birth, but some may present with subtle and nonspecific symptoms or may initially appear well. Among asymptomatic neonates, clinicians must identify those with bacteraemia and significant risks for progression to EOS. Previous studies have identified maternal risk factors (RFs) for EOS, and guidelines have suggested algorithms for managing asymptomatic neonates with RFs.[1] However, the majority of information for risk assessment was derived from data obtained before the widespread use of intrapartum antibiotic prophylaxis (IAP) for EOS prevention.[2] Recent data have reduced the validity of the risk-based approach,[3] which results in prolonged hospitalization and unnecessary antibiotic use for a large number of well-appearing infants.[4,5] The Centers for Disease Control and Prevention (CDC) guidelines recommend a full diagnostic evaluation and antibiotic therapy if a patient shows signs of sepsis, a limited evaluation and antibiotic therapy in cases of chorioamnionitis, and a limited evaluation and observation in cases of preterm birth or prolonged membrane rupture.[1] Two recent European guidelines recommend observation without further testing for all asymptomatic neonates with RFs,[6,7] although neither guideline provides any data to support their recommendations. However, neonatal management is controversial, especially among chorioamnionitis-exposed newborns.[1,8–10] Because EOS has become rare due to widespread use of IAPs and because chorioamnionitis is also uncommon (0.5–10% of deliveries),[8] large cohorts of newborns are better suited to providing information regarding the safety and efficacy of a given strategy.

We reviewed GBS-EOS cases (at or above 35 weeks of gestation) that occurred over a 14-year period (from 2003 to 2016) in an Italian population of over 530,000 live births. Hospital records of newborns with EOS were reviewed in detail to determine obstetrical RFs and the severity of neonatal disease according to the timing of symptom onset and IAP exposure. This analysis was conducted to evaluate the safety of a new Italian strategy involving serial physical examinations (SPEs) for the management of asymptomatic neonates at risk for EOS.[4,11,12] This strategy was introduced in the last months of 2009. Because SPEs were fully implemented beginning in 2010, EOS cases occurring before (2003–2009) and after (2010–2016) the implementation of this strategy were compared.

## Materials and methods

### Study design

A screening-based strategy (prenatal screening at 35–37 weeks of gestation) and IAP according to the CDC guidelines[1] are in place in Emilia-Romagna, which is a northern region of Italy.[13,14] Since 2003, a network of GBS active and area-based surveillance has included all regional birth facilities. Cases with a positive blood or cerebrospinal fluid (CSF) culture in an infant younger than 3 months of age are notified to the coordinating centre.[12] In order to minimize missed cases, an e-mail is sent on a monthly basis to all regional consultant paediatricians and microbiological laboratories to ask for notification. Demographics, modes of delivery, RFs for EOS, IAP administration and clinical information are obtained from the labour and delivery records by surveillance officers using a standardized form.[12] Incomplete data is retrieved via a telephone call from the coordinating centre.

This is a prospective, area-based and time-based cohort study involving regional birth facilities of Emilia-Romagna. Total cases of EOS that occurred from 1 January 2003 to 31 December 2016 in infants aged at or above 35 weeks of gestation were analysed in detail. To maintain patient confidentiality, spreadsheets submitted to the principal investigator were anonymous and did not include any data that would have allowed identification of patients or caregivers. Furthermore, cases of EOS that occurred from 2003 to 2009 (baseline period) were managed according to CDC guidelines. They were compared with cases that occurred from 2010 to 2016 (intervention period), when a new strategy for managing neonates (SPEs) was introduced. The project was approved by the local ethical committee of Azienda Ospedaliero-Universitaria di Modena, Italy (No 265/17).

### Definitions

*GBS early-onset disease*: GBS isolated from a normally sterile body site (e.g., blood or CSF) in infants between 0 and 72 hours old.

*Risk factors*: According to the CDC guidelines,[1] the RFs include preterm birth (<37 weeks of gestation), a previous infant with a GBS invasive infection, rupture of membranes (ROM) ≥18 hours prior to delivery, GBS bacteriuria identified during the current pregnancy and a maternal intrapartum fever ≥38°C during labour (as a surrogate for chorioamnionitis).

*At-risk newborn* is defined as an infant whose mother is GBS colonized or has one or more RFs for EOS.[4]

*Adequate IAP*: Penicillin, ampicillin or cefazolin given *i*.*v*. at least 4 hours prior to delivery.[1]

*Severe disease*: Includes any of the following: death, meningitis, seizures, brain lesions at hospital discharge, need for catecholamine support or mechanical ventilation.

### The management of well-appearing neonates (at or above 35 weeks of gestation)

From 2003 to 2009 (baseline period) neonates at risk of EOS exposed to inadequate IAP were managed according to CDC guidelines.[1] They had a CBC count, serial CRPs, and a blood culture after birth. Empirical treatment with antibiotics was given on the basis of clinical presentation, laboratory evaluation, and blood culture results. All neonates exposed to intrapartum fever (which we used as a surrogate for chorioamnionitis) were given empirical treatment with ampicillin and gentamicin until EOS was excluded.

Periodical meetings among centres attest that neonates at risk of EOS were managed from 2010 to 2016 (intervention period) through SPEs.[4,12] The need for laboratory testing or antibiotics was based on only the clinical presentation. All neonates were left in the rooms with their own mothers without being admitted to the NICU or level II nursery. Clinical monitoring was performed by midwives, bedside nursing staff and physicians. A standardized form signed by each examiner was used to detail general wellbeing, skin colour and respiratory signs at standard intervals (at ages 3, 6, 12, 18, 24, 36 and 48 hours). Every newborn with symptoms of suspected sepsis was immediately referred to a neonatal care specialist.

However, as far as concerns well-appearing neonates exposed to intrapartum fever, the approach was not uniform. Approximately 25% of the centres managed neonates through only SPEs, whereas the remaining 75% of the centres still obtained blood cultures and a WBC count at birth. Unlike the CDC guidelines,[1] clinician did not administer empirical antibiotics, unless neonates became symptomatic, had abnormal laboratory results, or blood culture yielded pathogens.

### Statistical analyses

The analyses were performed using STATA/SE 14.2 for Windows and MedCalc version 9.3 (MedCalc Software, Ostend, Belgium). Continuous data were reported as the means±SDs, and categorical variables were reported as numbers and percentages. Incidence rates were calculated as cases per 1000 live births. The total number of live births was provided by the Regional Health Agency.

## Results

### Overall population

During the 14-year study period, 532,154 live births (at or above 35 weeks of gestation) occurred in the region (266,646 from 2003 to 2009 and 265,508 from 2010 to 2016). Surveillance identified 108 EOS cases at or above 35 weeks of gestation, of which 99 (91.7%) were born full-term (≥ 37 weeks of gestation) and 9 (8.3%) were born preterm (35–36 weeks of gestation). Demographic and clinical data of newborns with EOS are shown in Table 1 according to study period. Most neonates with EOS were born to pregnant women who had a negative prenatal screening test or were not screened. However, rates of false-negative prenatal screening had a borderline decrease in 2010–2016. Furthermore, rates of IAP exposure as well as age at onset of symptoms differ significantly in study period. In contrast rates of meningitis, mechanical ventilation and mortality did not change in study period. Most neonates with EOS were not at risk and were unexposed to IAP.

**Table 1:** Demographic and clinical data of neonates with EOS at or above 35 weeks of gestation according to study period.

	Cases of EOS in 2003–2009 (n = 60)	Cases of EOS in 2010–2016 (n = 48)	*p*	Total cases of EOS (n = 108)
Incidence per 1000 live births *(C*.*I*.*)*	0.23 (0.2284–0.2315)	0.18 (0.1785–0.1814)	0.26	0.20 (0.1989–0.2010)
Birth weight, *median*, *g (IQR)*	3240 (3010–3630)	3351 (3025–3570)	0.86	3270 (3011–3600)
Gestational age at delivery, *median*, *weeks (IQR)*	39.0 (38.0–40.0)	39.0 (38.0–40.0)	0.92	39.0 (38.0–40.0)
Prenatal screening, *n (%)*	52 (86.7)	37 (77.1)	0.30	89 (82.4)
Positive prenatal screening, *n (%)* ^*a*^	15 (28.8)	19 (51.4)	0.05	34 (31.5)
At least 1 risk factor (except GBS positive screening), *n (%)*	21 (35.0)	18 (37.5)	0.90	39 (36.1)
No RF, GBS screening negative or unknown, *n (%)*	31 (51.7)	16 (33.3)	0.09	47 (43.5)
IAP exposure, *n (%)*	7 (11.7)	16 (33.3)	0.01	23 (21.3)
Mechanical ventilation, *n (%)*	4 (6.7)	10 (20.8)	0.06	14 (13.0)
Meningitis (±sepsis), *n (%)* ^*b*^	6 (10.0)	1 (2.1)	0.12	7 (6.5)
Mortality, *n (%)*	0	1 (2.1)	0.92	1 (0.9)
Severe disease, *n (%)*	7 (11.7)	10 (20.8)	0.30	17 (15.7)
Age at presentation, *median*, *hours (IQR)* ^*c*^	6 (1–18.7)	1 (0.0–4.0)	0.01	4 (0.0–12.0)

**C.I.,** confidence interval; **EOS,** early-onset sepsis; **GBS**, group B streptococcus; **IAP**, intrapartum antibiotic prophylaxis; **IQR**, interquartile range; **RF**, risk factor for early-onset sepsis

^*a*^ Percentage and significance are calculated based on the women who underwent antenatal screening in both periods

^*b*^ 34 of 60 neonates (2003–2009) and 23 of 48 neonates (2010–2016) did not undergo lumbar puncture; the significance of meningitis is calculated based on the neonates who underwent lumbar puncture in both periods

^*c*^ Asymptomatic neonates were excluded from calculation

### Asymptomatic or symptomatic cases of EOS

**Fig 1:**
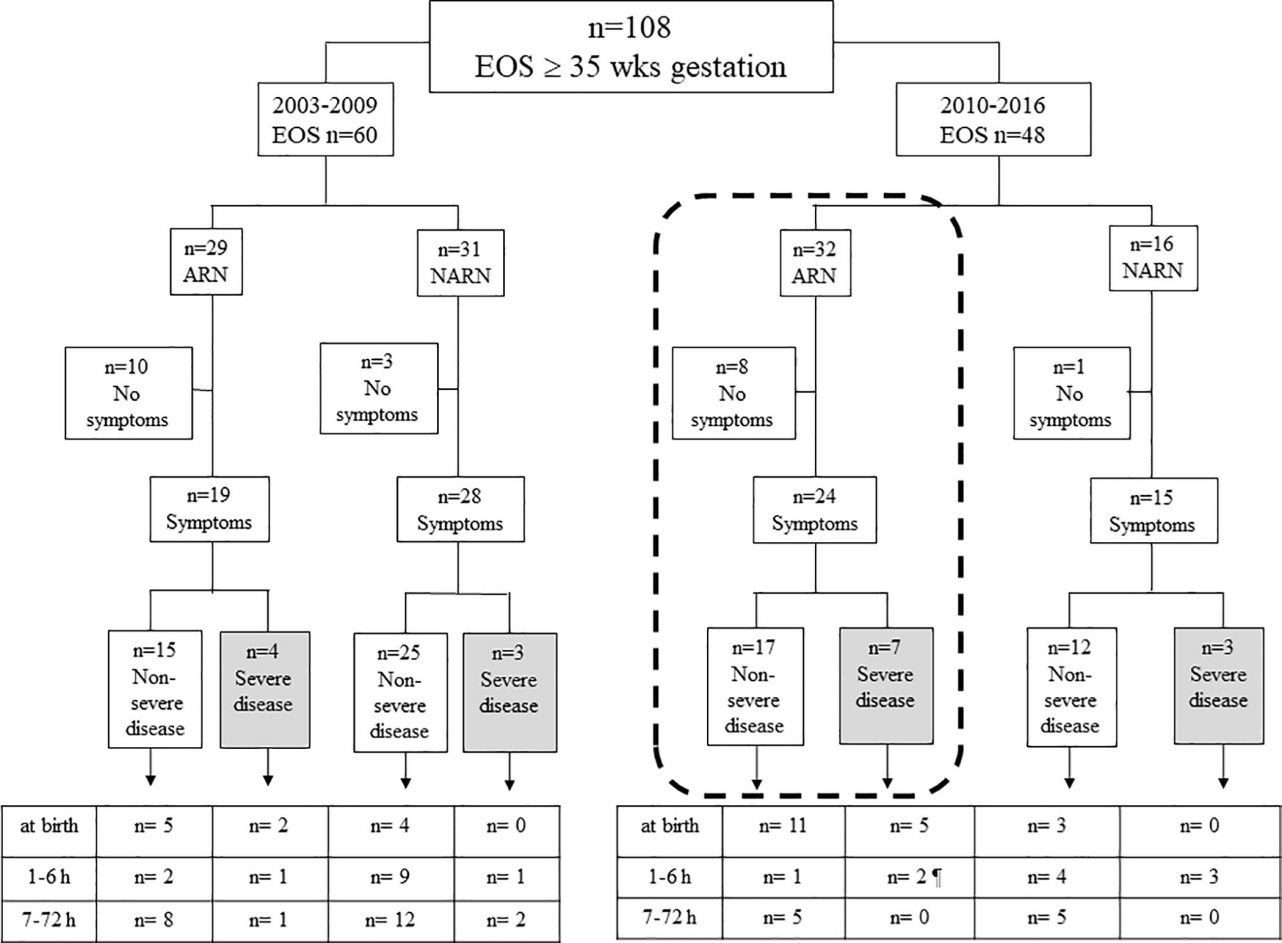
EOS cases (at or above 35 weeks of gestation) in the 2 study periods (2003–2009 vs 2010–2016). Cases are divided according to the presence (or absence) of risk factors, the presence (or absence) of symptoms during the first 72 hours of life and the time of onset of symptoms. Cases of severe disease are shown in the grey boxes. Neonates managed through SPEs are shown in the dashed box.ARN, at-risk neonates; EOS, early-onset disease; h, hours; NARN, not at-risk neonates; wks, weeks. ¶ One neonate with severe disease was unexposed to IAP; he was born preterm (36 weeks of gestation) to an unscreened mother, and symptoms developed at 2 hours of age. Another neonate was treated in labour with 8 doses of clindamycin; he was born after a prolonged membrane rupture (60 hours) and symptoms developed at 4 hours of age.

Fig 1 shows the 108 EOS cases. The presence (or absence) of symptoms during the first 72 hours of life, disease severity and age at presentation are shown for both study periods (2003–2009 and 2010–2016), during which timeframes different strategies for managing neonates were adopted. The boxes at the bottom of the Fig 1 show the ages of newborns at the onset of symptoms.

Twenty-two (20.4%) EOS cases (13 from 2003 to 2009 and 9 from 2010 to 2016) remained asymptomatic through the first 72 hours of life. The need for laboratory testing and blood culture after birth was the presence of one or more RFs in 18 of 22 neonates (exposed to intrapartum fever: n = 10). The remaining 4 asymptomatic neonates, who were not at risk, were sampled because of umbilical cord donation (n = 2) or unknown reason (n = 2).

Eighty-six (79.6%) neonates (47 from 2003 to 2009 and 39 from 2010 to 2016) were symptomatic. Thirty neonates developed symptoms at birth. The remaining 56 neonates developed symptoms from 1 to 72 hours of life, and most of them were not at risk for EOS.

### EOS cases managed (from 2010) through SPEs

Thirty-two neonates were at-risk for EOS and were managed (from 2010) through SPEs. They are shown in the dashed box in Fig 1.

Eight newborns (25.0%) were investigated because of intrapartum fever. They remained asymptomatic. All were given *i*.*v*. antibiotics once blood culture results were available. Some of them started treatment over age 24 hours.

Twenty-four neonates developed symptoms. Seven neonates had severe disease. Five of them developed symptoms at birth; 1 developed symptoms at 2 and 1 at 4 hours of life respectively (their details are given in the footnote of Fig 1). The remaining 17 neonates had non severe disease, of which 5 developed symptoms from 7 hours onward (at 7, 9, 14, 24 and 48 hours respectively). Two of those five newborns had the most delayed presentation of symptoms (jaundice at 24 and 48 hours respectively); both were unexposed to IAP.

### Symptomatic neonates exposed or unexposed to IAP

**Fig 2:**
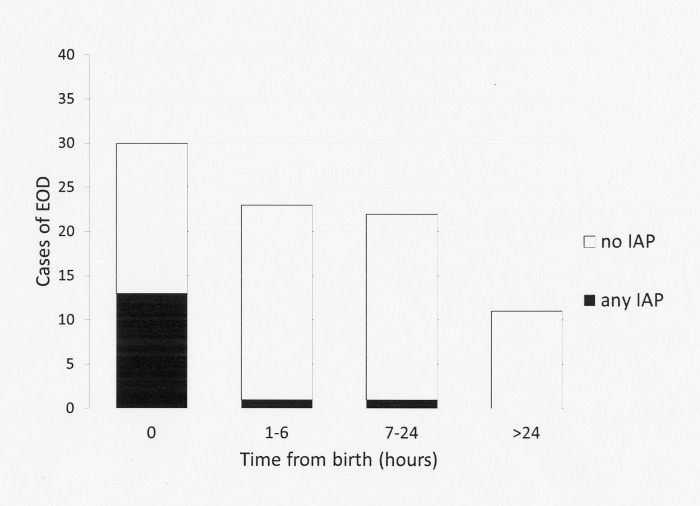
Age of onset of symptoms among IAP-exposed and unexposed neonates (asymptomatic neonates are excluded). IAP, intrapartum antibiotic prophylaxis. Empty bars: cases unexposed to IAP. Black bars: cases exposed to any IAP. Symptoms developed at 0 hours (beta-lactam antibiotics ≥4 hours, n = 4; beta-lactam antibiotics <4 hours, n = 4; non-beta-lactam antibiotics, n = 5). Symptoms developed at 1 to 6 hours (non-beta-lactam antibiotics, n = 1). Symptoms developed at >6 hours (beta-lactam antibiotics ≥4 hours, n = 1).

Among the 86 neonates with symptoms, the median age at presentation was 4 hours (IQR 0.0–12.0). With respect to the IAP-unexposed neonates, the median age at presentation was 6 hours (IQR 1.0000–15.0000). IAP-exposed neonates were more likely to present earlier, with a median age at presentation of 0 hours (IQR 0.0000–0.0000, p<0.001). Fig 2 shows the age of symptom onset among the IAP-exposed and unexposed neonates. The antibiotics administered in each case are shown in the footnotes. Only one IAP-exposed newborn developed EOS symptoms (a mild tachypnoea) more than 6 hours after birth.

### Neonates exposed to intrapartum fever

Eighteen neonates were exposed to intrapartum fever, of which 10 (55.6%) remained asymptomatic through the first 72 hours of life. The remaining 8 neonates (44.4%) developed symptoms (at birth, n = 7; or at age 7 hours, n = 1).

The expected number of pregnant women with intrapartum fever (n = 5854) was calculated according to the prevalence of intrapartum fever (1.1%) in our population.[13] By assuming full implementation of the CDC guidelines, the number of newborns potentially treated (number needed to treat, NNT) for each infant asymptomatic at age 0 to 6 hours with culture-proven EOS was calculated according to Wortham.[8] The NNT was 531 (Table 2). EOS was confirmed in only 0.31% of infants exposed to intrapartum fever.

**Table 2:** Number of neonates needed to treat for each neonate asymptomatic at age 0 to 6 hours who was exposed to intrapartum fever.

Variables	*n*
Total number of live births at or above 35 weeks of gestation	532,164
Prevalence of intrapartum fever among pregnant women (%)	1.1
Number of neonates exposed to intrapartum fever	5854
Number of neonates with symptoms at age 0 to 6 hours	7
Number of asymptomatic neonates	5847
Number of neonates asymptomatic at age 0 to 6 hours with a positive blood culture	11
NNT	531

**NNT**, Number needed to treat

## Discussion

This study shows the clinical impact of a 14-year screening-based policy[12,13] for EOS prevention in an Italian cohort and the results of a strategy for managing asymptomatic neonates at risk. A significant proportion of newborns with EOS were at low risk for sepsis. Most cases were unexposed to IAP because they were born to pregnant women who tested negative for GBS colonization or were not tested as they were not at risk. High rates of neonates with EOS born to GBS-negative mothers have been reported in areas where the screening-based strategy and IAP reached high coverage,[15,16] which is one of the main drawbacks of this strategy. However, the rates of false-negative screening cultures had a borderline decrease over time.

Guidelines for prevention have led to a decline in EOS worldwide, although they have resulted in laboratory evaluations and antibiotic exposure for numerous well-appearing uninfected neonates.[17]

A recent review estimates that 14% of full-term neonates in the European Union are evaluated and that 8% are treated with antibiotics annually, although only 0.1% have proven EOS.[5] Therefore, alternative strategies for managing neonates are warranted. The SPEs strategy has changed dramatically the management of well-appearing neonates at-risk for EOS in 2 studies performed in our region. The number of well-appearing newborns undergoing laboratory testing decreased from 11.6% to 1.6% (p < 0.01) in a single centre study.[4] In a subsequent, retrospective study carried out in 3 NICUs of Emilia-Romagna, only 3.0% of initially asymptomatic neonates at risk for EOS were evaluated.[18]

We reviewed EOS cases from a large cohort of newborns in detail. Most cases with symptoms that occurred after birth were not at risk for EOS. Cases among neonates at risk were diagnosed in a timely manner after 2010 through SPEs, and none among those who developed symptoms after 6 hours of life had severe disease. Two cases of severe disease developed symptoms at 2 and 4 hours of life. However, with such an early symptom onset, no strategy, including laboratory testing or empirical antibiotic therapy at birth, could have mitigated their severity. This very close observation during the first hours of life seems an excellent tool to timely diagnose symptoms of EOS, likely before common laboratory testing results. Indeed, rates of meningitis, mortality and mechanical ventilation did not change in study periods.

IAP exposure increased significantly in 2010–2016, perhaps because of the decrease of false-negative screening cultures. IAP-exposed neonates with confirmed EOS were more likely to be symptomatic at birth than unexposed neonates. This finding is reassuring for clinicians who manage IAP-exposed neonates who appear well at birth. The risk of developing symptoms after 6 hours of life was extremely low, because only one neonate became symptomatic. In contrast, a number of IAP-unexposed newborns developed symptoms many hours after birth. One possible explanation for this finding is that IAP exposure reveals newborns who have developed an advanced infection in utero (who are symptomatic at birth) while protecting neonates from the EOS that would be acquired during passage through the birth canal. In addition, only 0.31% of newborns exposed to intrapartum fever had confirmed EOS. Thus, the NNT to prevent one case among asymptomatic neonates was high (>530) and might have been even higher, because only one among the initially asymptomatic neonates with confirmed EOS developed symptoms after 6 hours of life.

The safety and efficacy of SPEs for managing chorioamnionitis-exposed newborns was tested recently in 277 well-appearing chorioamnionitis-exposed neonates (≥34 weeks of gestation) in a single US centre.[19] The authors found that the SPE strategy was associated with a 55% reduction in antibiotic exposure without any adverse outcomes. Since many infants with EOS have a low risk of sepsis, the authors recommend that the SPE strategy should include all asymptomatic newborns regardless of the presence of RFs for EOS.[19]

Our data, obtained from a large cohort of newborns, attest to the safety of an observation-based approach for managing all asymptomatic newborns at risk for EOS, although definite conclusions on causation are lacking. Furthermore, we do not know whether this strategy reduces unnecessary antibiotic use compared to other strategies, while remaining equally safe. In the USA, the neonatal sepsis calculator (NSC) provides an infant’s risk score through an evidence-based algorithm derived from perinatal parameters (gestational age, duration of membrane rupture, highest maternal temperature during labour, group B streptococcal colonization status, and IAP) and the neonatal examination. In a US cohort study that included 56,261 live births at or above 35 weeks of gestation, empirical antibiotic use decreased from 5.0% to 2.6% for the neonates.[10] Strunk and co-workers reported that after implementation of the NSC in a single centre in Australia, the proportion of infants treated with antibiotics decreased from 12.0 to 7.6%.[20] In contrast, the above mentioned study performed in three Italian centres showed that empirical antibiotics were given to 1.4% of the entire cohort of neonates after implementation of the SPE strategy.[18] Whether these differences between the NSC in the USA and Australia and the SPE strategy in Italy depend on the different populations is unclear, and further studies comparing both strategies are required to answer this question.

Our study is subject to important limitations. Firstly, this study is a comparison of time-based cohorts, as infants were not randomized to each strategy, and our results would not be generalizable. In addition, we have no data regarding neonatal antibiotic exposure after SPEs in the entire region, although in a single centre study antibiotic exposure was decreased by over 4 times.[4] A prospective cohort study to evaluate current rates of antibiotic exposure in the whole region is ongoing. Furthermore, the approach used to manage neonates exposed to intrapartum fever was not uniform among our regional centres because some centres still obtained laboratory testing results. This finding may reflect current uncertainties regarding both a clear definition of chorioamnionitis and a safe management of these high risk newborns. However, a substantial proportion (25%) of neonates exposed to intrapartum fever were managed from 2010 to 2016 through SPE alone, without any adverse outcome regardless of risk. Based on this experience, all regional centres currently adopt the SPE strategy for managing all asymptomatic newborns at ≥34 weeks of gestation.

In conclusion, this large cohort study evaluates risk factors for EOS, the presence of symptoms during the first 72 hours of life and the time of onset of symptoms according to IAP exposure. IAP-exposed neonates who appear healthy at birth have a very low chance of having EOS. Rates of confirmed EOS among neonates exposed to intrapartum fever are low. Furthermore, we report the results of a new strategy for managing neonates with RFs for EOS. Although definite conclusions on causation are lacking, our data support SPEs of asymptomatic newborns with EOS risk factors. This strategy may be a safe and effective alternative to laboratory screening tests and empiric antibiotic therapy.

## Supporting information

S1 File Early onset sepsis 2003–2016. (XLS)
